# Knowledge, attitudes and use of labour analgesia among women at a low-income country antenatal clinic

**DOI:** 10.1186/s12871-015-0078-9

**Published:** 2015-07-07

**Authors:** Mary T. Nabukenya, Andrew Kintu, Agnes Wabule, Mark T Muyingo, Arthur Kwizera

**Affiliations:** 1 Department of Anaesthesia and Critical Care, Makerere University and Mulago National Refferal Hospital, Kampala, Uganda; 2 Department of Obstetrics and Gynaecology, Makerere University College of Health Sciences, Kampala, Uganda

**Keywords:** Labour Analgesia, Perceptions, Low-and-middle income country, Uganda

## Abstract

**Background:**

Childbirth is one of the most painful experiences of a woman’s life. Authorities in the fields of obstetrics and anaesthesia encourage use of labour analgesia. Unlike in high-income countries, pain relief in labour in Africa is not a well established service, especially in the low-income countries like Uganda. Little is known about whether parturients would be amenable to labour analgesia. We sought to determine knowledge, attitudes and use of labour analgesia among women attending the antenatal clinic at Mulago National Referral Hospital.

**Methods:**

Upon obtaining institutional approval, we conducted a cross-sectional descriptive study. Women were requested to complete the researcher-administered survey following informed consent. The study was conducted in the general antenatal clinic at the Mulago National Referral Hospital.

**Results:**

Of 1293 participants interviewed, only 7 % of the participants had knowledge of labour analgesia. Of the multiparous mothers 87.9 % did not have labour analgesia in their previous deliveries, although 79.2 % of them had delivered in a national referral hospital. The commonest reason for refusal of labour analgesia was to experience natural childbirth. 87.7 % of the participants wanted labour analgesia for their next delivery.

**Conclusion:**

There is a wide gap between the desire for labour analgesia and its availability. Obstetricians and anaesthesiologists have a role to educate the women, and to provide this much desired service.

## Background

The pain of childbirth has been documented to be extreme. A lot of controversy has existed since the inception of pain relief in labour to date. According to the American Society of Anesthesiologists (ASA) and American College of Obstetricians and Gynecologists (ACOG), maternal request represents sufficient justification for pain relief [1, 2]. The American College of Obstetricians and Gynecologists also states that ‘labour results in severe pain for many women. There is no other circumstance where it is considered acceptable for a person to experience untreated severe pain, amenable to safe intervention, while under a physician's care’ [3].

In a bid to attain Millenium Development Goals 4 and 5 [4], attention is being focused on the very important area of childbirth. Analgesia for labour is widely utilized in high-income countries but this is not the case in Africa [5]. Issues in high-income countries are focused on the choice of methods and complications, while in developing countries the issue revolves around awareness, acceptability and availability of analgesia for labour [6].

Various studies have been conducted on the subject not only in low income countries [6–14], but also in high income countries [15] and they have shown various factors affect women’s attitudes to pain relief in labour. These include knowledge of labour analgesia (found to be low in several studies [6–8, 10–12, 14], upbringing and culture [7, 16], education level [6, 11, 12, 17], age, among others.

Currently, the method of analgesia available in the labour ward is continuous support, which is inconsistently offered, mainly by the understaffed midwives. There being little data and no protocols for pain relief in labour in our setting, the study sought to assess the participants’ knowledge of pain relief during labour and their beliefs, values and attitudes toward labour analgesia.

## Methods

Upon obtaining institutional and ethical approval from the Makerere University College of Health Sciences School of Medicine Research and Ethics Committee, we performed a cross-sectional descriptive study.

The study was conducted in Mulago National Referral Hospital, which is also the teaching hospital of Makerere University. It serves a variable population from all-over the country, and also handles referrals from neighbouring countries. It was carried out in the general antenatal clinic in the hospital.

The clinic runs 3 days every week. Over 1,000 mothers are seen on average monthly, of which 400–500 are attending their first antenatal visit. The first antenatal visit for most of the participants was during the first trimester, although a few of them attended their first antenatal clinic during the second or third trimesters.

Women were requested to complete the researcher-administered survey following informed consent. The mothers read the survey and study investigators were available to assist participants completing the survey and clarify any questions that arose. Verbal and written Luganda (the common local dialect) translation was provided to those whose first language was Luganda. The survey was conducted in the morning when study participants were still fresh.

The survey consisted of two sections. The first section examined demographics, (age, religion, education level, parity, occupation, residence and previous caesarean delivery). The second section consisted of 15 questions, and assessed knowledge and options of labour analgesia, sources of information and perceptions on labour analgesia. Among those with a previous experience of delivery, participants were to score their previous pain experience using the numeric rating score. On average, each survey took about 10 min. These questions were developed from a focused discussion by the authors. Each question expressed only one idea (i.e., no questions contained “and”) and no questions were phrased in a negative form. Answer types included choosing from a menu of choices, yes/no/neutral, or pains scores on a scale of 0 to 10 (not and).

### Statistical analysis

Calculation of the sample size was based on the number of factors we wanted to analyze as possible predictors of the use of labour analgesia during labour. In order to evaluate up to 10 predictive factors in a multivariate model and ensure stability of the regression calculation, we estimated the requirement of 120 patients with the outcome measure per degree of freedom. Thus, a minimum of 1,200 patients would be required.

Descriptive statistics were used to summarize demographics and outcomes. We first conducted univariate analyses (Student -test, Wilcoxon-Mann–Whitney or Chi-squared tests as appropriate) to analyze factors determined a priori to be potentially important. A factor found to have a statistically significant association with a patient having no knowledge of labour analgesia with univariate analysis was considered a potential predictive factor. Data were analyzed using Microsoft Excel and SPSS (Version 11).

## Results

**Fig. 1:**
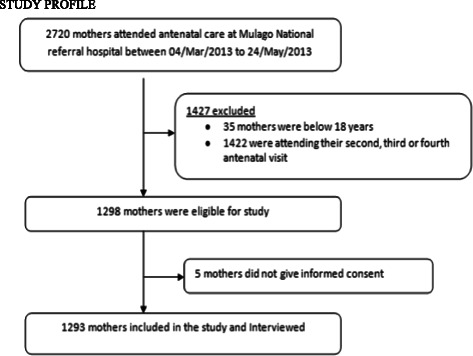
Study profile

A total of 1,293 pregnant women was recruited in this study out of 2,720 mothers who attended the antenatal clinic during the study period (Fig. 1). Of these, 66 % were 30 years and below, 80 % had at least one child and 55 % had attained secondary education and above (Table 1).

**Table 1:** Demographics

Characteristic	Distribution of study participants,	
	Total = 1,293	
	Number	Percentage
Age groups in years		
1. 18–30	850	65.8
2. 31–40	429	33.2
3. >40	12	0.9
4. No response	2	0.1
Religion		
1. Catholic	405	31.3
2. Anglican	381	29.5
3. Muslim	328	25.4
4. Others	175	13.5
5. No response	4	0.3
Education Level		
1. No education	29	2.2
2. Primary	546	42.2
3. Secondary	624	48.3
4. Undergraduate	81	6.3
5. Post graduate	8	0.6
6. No response	5	0.4
Occupation		
1. Unemployed	554	42.8
2. Small business	597	46.2
3. Professional	93	7.2
4. Informal	49	3.8
Residence		
1. Rural	122	9.4
2. Urban	1,165	90.1
3. No response	6	0.5
Parity		
1. Primiparous	264	20.4
2. Multiparous	1,029	79.6
Previous C/S		
1. Yes	503	38.9
2. No	788	60.9
3. No response	2	0.2

Only 91 (7 %) of the women in this study had any knowledge of labour analgesia (Table 2).

**Table 2:** Knowledge of Labour Analgesia

	Number	Percentage	95%CI
Yes	91	7.0	5.64–8.43
No	1,202	93.0	91.57–94.36

**Fig. 2:**
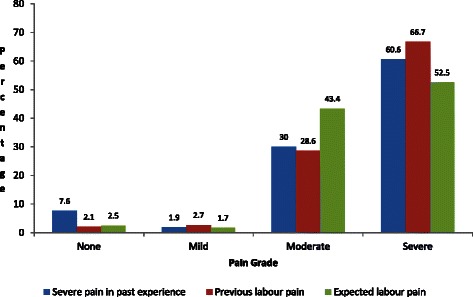
Pain scores

Regarding attitudes and beliefs, 87.8 % of the participants felt that labour should be pain-free, 10 % that labour pain is natural and should be experienced. Of those with experience of previous labour, 686 (66.7 %) described the pain as severe (Fig. 2). Participants who thought any doctor could give labour analgesia were 967 (78.6 %), and 3 (0.2 %) said an anaesthesia provider gives labour analgesia. All three were multiparous. A significant majority, 1,134 (87.7 %) of the participants said they wanted to have labour analgesia for their next delivery (Table 3). However, even among those who wanted labour analgesia for the next delivery, there were some concerns, mainly that the baby may be affected (54.5 %), the method may not work (23.4 %), among others (Table 3).

**Table 3:** Desire for labour analgesia, place of previous delivery, and concerns about labour analgesia

Variable	Distribution of participants	
	Number	Percentage
Do you want labour analgesia? (Total 1293)		
1. Yes	1,134	87.7
2. No	156	12.1
3. No response	3	0.2
Place of previous baby delivery among the parous (Total 1029)		
1. Private health facility	181	17.6
2. National referral hospital	815	79.2
3. No response	33	3.2
Concerns about pain relief		
1. Baby may be affected	391	54.5
2. Contractions may be weakened	45	6.3
3. Inability to push or use lower part	68	9.5
4. May lead to C/S or instrument use	5	0.7
5. Method may not work	168	23.4
6. Other	41	5.7

**Fig. 3:**
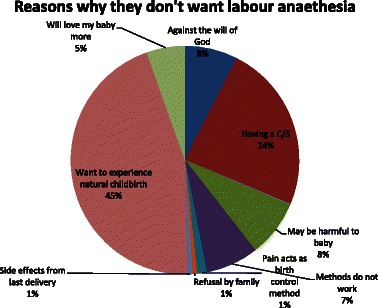
Among those who do not want labour analgesia

Concerning those who did not want labour analgesia for their next delivery, 45 % said they wanted to experience natural childbirth, 8 % said it was against the will of God, 8 % thought it would harm the baby, 5 % said they would love their baby more, 1 % said the pain was a form of birth control (Fig. 3).

**Fig. 4:**
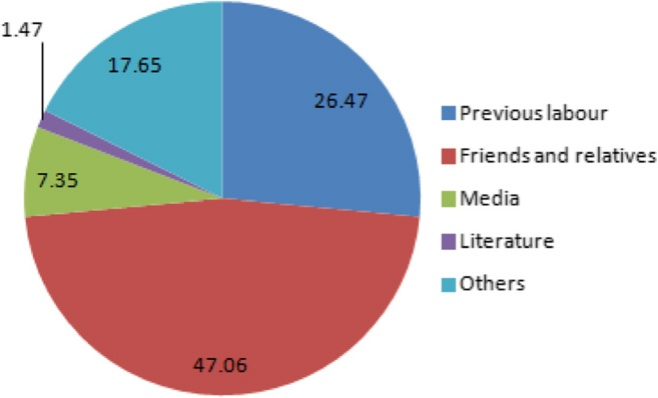
Source of Information about Labour Analgesia

**Fig. 5:**
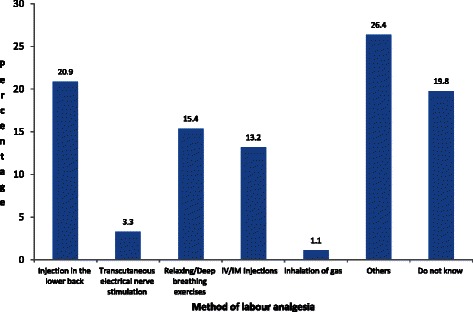
Known methods among those who had knowledge of labour analgesia

Majority (47 %) of those who knew about labour analgesia got the information from friends and family,26 % from the previous labour, 7 % from the media and 1 % from literature (Fig. 4). Among methods of analgesia known, 24 (26 %) women said local herbs (“others” in Fig. 5), 19 (21 %) injection in the lower back (whether spinal or epidural), among others (Fig. 5). No mother mentioned “epidural” in particular, however. The study showed multiparity had a positive correlation (0.52 CI 0.32–0.85, *p*-value 0.009) with acceptability of labour analgesia, while age and education did not affect acceptability (*p*-values > 0.08). Also, those who had history of Caesarean section were less likely to accept labour analgesia (1.88 CI 1.34 – 2.63, *p*-value 0.001).

Asked if any method of labour analgesia had been used in the previous delivery, 894 (87.9 %) of the participants reported that they did not get labour analgesia, although 79.2 % of the multiparous participants had delivered in the national referral hospital (Table 3).

## Discussion

This study found that very few mothers knew of labour analgesia. Despite their lack of prior knowledge, majority want to have labour analgesia for their next delivery. Of the multigravida mothers, many had delivered in a national referral hospital, however many said they did not have labour analgesia during their previous delivery. Among those who had knowledge of labour analgesia, the commonest source of information was friends and relatives. Few got information from the previous labour, even fewer from media and literature. While over 50 % had attained at least secondary education, the number with primary level education and below is still significant.

Many of the multigravida mothers described the pain from their previous delivery as severe. It is therefore not surprising that majority of the mothers in the study (both prime gravida and multigravida) believed that labour should be pain-free. However, a few mothers did not want labour analgesia for their subsequent delivery and the commonest reason was to experience natural childbirth; others said it was against the will of God.

From these results, there is a clear need for labour analgesia by the pregnant mothers, however for various reasons, it is not provided. One of the contributing factors is the fact that there is no established labour analgesia service, even in the national referral hospital. Given the commonest source of information, the mothers are not getting this information from the obstetricians during their antenatal visits. A very small percentage of mothers cited literature as a source of information because the literature is not readily available. Judging from the various beliefs and views expressed by the mothers regarding labour analgesia, culture and religion play a role in decisions made. The results also showed that multigravida mothers are more likely to accept labour analgesia.

Several studies have been done on the subject in other developing countries. The study by Naithani et al. is comparable to this study (9.5 % knowledge of labour analgesia) [11]. Many other studies in LMICs also showed low level of knowledge [6–8, 10] compared to the developed world [18]. An audit by Taneja, Nath and Dua (2004) in India showed that majority of the obstetricians were not taught labour analgesia during their training programme and their practical exposure to it was very limited [19]. This may be the case in this study as well. Regarding acceptability of labour analgesia, this study was comparable to that by Audu et al. [7]. However, acceptance was not high in the studies by Naithani et al. [11] and Olayemi et al. [6], the commonest reason being to experience natural childbirth, similar to this study. In the study by Toledo et al., desire to experience unmedicated (“natural”) childbirth was one of the major reasons for avoiding neuraxial analgesia [15]. Also similar to this study were the concerns about effects on the baby and the method leading to caesarean delivery. While this study showed a positive correlation between parity and acceptability of labour analgesia, Okeke et al. [12] found no association. Olayemi et al. [6] found that education had a positive correlation and age a negative correlation. In this study, age and education did not affect acceptance. No other studies were found comparing previous Caesarean section history and acceptance of labour analgesia. Our study showed a negative correlation. Some of the women who had Caesarean sections had long periods of labour pain before an emergency C-section was performed, and therefore would not want to go through labour again.

Our study limitations included the fact that we excluded mothers below 18 years and yet these contribute significantly to the prime gravida population in the hospital, and the country as a whole. It would have been worthwhile to investigate their attitudes and views concerning labour analgesia. Additionally, study was done in the antenatal period. It would probably be more informative if it was extended to and after the time of labour.

Having been conducted in the antenatal period, the study made no provision for analgesia at the time of labour. This is partly because it is not an established service in the hospital, hence the need for studies such as this one to form a baseline for establishing a labour analgesia service in the hospital. The standard of care is however offered to every parturient.

## Conclusion

From this study, it is very clear that there is a wide gap between desire for labour analgesia and its provision. Obstetricians and anaesthesia providers have a great role to play in educating the mothers, and possibly their colleagues, on the various methods of labour analgesia before the service can be set up. Labour analgesia is a standard of care in obstetrics and so should be provided in the national referral hospital.

In conclusion, very few pregnant mothers know about labour analgesia but majority would love it.

## Abbreviations

ACOG: American College of Obstetricians and Gynecologists
ASA: American Society of Anesthesiologists
LMICs: Low and Middle Income Countries
